# Ion channel profiles of extraocular motoneurons and internuclear neurons in human abducens and trochlear nuclei

**DOI:** 10.3389/fnana.2024.1411154

**Published:** 2024-06-18

**Authors:** Ümit S. Mayadali, Christina A. M. Chertes, Inga Sinicina, Aasef G. Shaikh, Anja K. E. Horn

**Affiliations:** ^1^Chair of Vegetative Anatomy, Faculty of Medicine, Institute of Anatomy, LMU Munich, Munich, Germany; ^2^Faculty of Medicine, Institute of Legal Medicine, LMU Munich, Munich, Germany; ^3^Department of Neurology, University Hospitals, Cleveland VA Medical Center, Case Western Reserve University, Cleveland, OH, United States

**Keywords:** eye muscles, voltage-gated potassium (Kv) channels, hyperpolarization-activated cyclic nucleotide-gated cation channels (HCN), perineuronal nets, singly-innervated muscle fibers, multiply-innervated muscle fibers

## Abstract

**Introduction:**

Extraocular muscles are innervated by two anatomically and histochemically distinct motoneuron populations: motoneurons of multiply-innervated fibers (MIF), and of singly-innervated fibers (SIF). Recently, it has been established by our research group that these motoneuron types of monkey abducens and trochlear nuclei express distinct ion channel profiles: SIF motoneurons, as well as abducens internuclear neurons (INT), express strong Kv1.1 and Kv3.1b immunoreactivity, indicating their fast-firing capacity, whereas MIF motoneurons do not. Moreover, low voltage activated cation channels, such as Cav3.1 and HCN1 showed differences between MIF and SIF motoneurons, indicating distinct post-inhibitory rebound characteristics. However, the ion channel profiles of MIF and SIF motoneurons have not been established in human brainstem tissue.

**Methods:**

Therefore, we used immunohistochemical methods with antibodies against Kv, Cav3 and HCN channels to (1) examine the human trochlear nucleus in terms of anatomical organization of MIF and SIF motoneurons, (2) examine immunolabeling patterns of ion channel proteins in the distinct motoneurons populations in the trochlear and abducens nuclei.

**Results:**

In the examination of the trochlear nucleus, a third motoneuron subgroup was consistently encountered with weak perineuronal nets (PN). The neurons of this subgroup had -on average- larger diameters than MIF motoneurons, and smaller diameters than SIF motoneurons, and PN expression strength correlated with neuronal size. Immunolabeling of various ion channels revealed that, in general, human MIF and SIF motoneurons did not differ consistently, as opposed to the findings in monkey trochlear and abducens nuclei. Kv1.1, Kv3.1b and HCN channels were found on both MIF and SIF motoneurons and the immunolabeling density varied for multiple ion channels. On the other hand, significant differences between SIF motoneurons and INTs were found in terms of HCN1 immunoreactivity.

**Discussion:**

These results indicated that motoneurons may be more variable in human in terms of histochemical and biophysiological characteristics, than previously thought. This study therefore establishes grounds for any histochemical examination of motor nuclei controlling extraocular muscles in eye movement related pathologies in the human brainstem.

## 1 Introduction

Lateral rectus and superior oblique muscles govern horizontal and torsional eye movements, respectively. While each of these muscles are innervated by a single motor nucleus of the cranial nerves, the trochlear (nIV) and abducens nucleus (nVI), respectively, the remaining four extraocular muscles are innervated by the oculomotor nucleus (nIII). As for all extraocular muscles, the axons of the abducens and trochlear nerves make contact with specific muscle fiber types in lateral rectus and superior oblique muscles by different types of axon terminals and innervation patterns: Fast-contracting, fatigable twitch-type muscle fibers are innervated by a single “en plaque” ending near the middle of the muscle belly (singly-innervated muscle fiber—SIF), whereas slow contracting, non-fatigable non-twitch type muscle fibers are targeted by multiple “en grappe” nerve endings (multiply-innervated muscle fiber—MIF) along the entire length of the fiber (for review: Horn and Straka, 2021).

Further, extraocular muscles consist of two layers. Only muscle fibers of the global layer insert on the bulbus via a tendon and initiate the eye movements, whereas the muscle fibers of the outer orbital layer insert on the connective tissue openings of Tenon‘s capsule forming pulleys, which act as muscle origin and activate later (Demer et al., 2006). Both layers differ in their SIF and MIF content, as global layer consists of approximately 90% SIFs and 10% MIFs, and orbital layer consists of approximately 80% SIFs and 20% MIFs (Spencer and Porter, 2006). However, recent discoveries suggest that extraocular muscle layers are not innervated by anatomically separated motoneuron groups and it is therefore not likely that these layers function independently (Bohlen et al., 2017; Adade and Das, 2023).

Despite the dual classification into MIFs and SIFs based on their innervation pattern, at least 11 distinct myosin isoforms are differentially expressed in MIFs and SIFs of the global and orbital layer (Hoh, 2021). The variety of dynamics in eye movements may be achieved through recruitment of various sets of these muscle fiber types by corresponding subsets of motoneurons that innervate these fibers (Horn and Straka, 2021).

Tracer injection into the distal tendons of lateral rectus and superior oblique muscles and consequent selective tracer uptake by the “en grappe” endings results in retrograde labeling of only MIF motoneurons in the abducens (nVI) and trochlear nuclei (nIV), respectively (Büttner-Ennever et al., 2001; Büttner-Ennever, 2006). In monkey brainstem, these MIF motoneurons are located in the medial-dorsal periphery in the abducens nucleus, interspersed with SIF motoneurons, and in a dorsal cap of the trochlear nucleus, separated from most SIF motoneurons (Büttner-Ennever et al., 2001; Büttner-Ennever, 2006). Within the abducens nucleus, another population, the non-cholinergic internuclear neurons (INT) relay pre-motor signals to the motoneurons of the medial rectus muscles in the contralateral oculomotor nucleus for synergistic eye movements on the horizontal plane (Büttner-Ennever et al., 1981; Fuchs et al., 1988).

MIF motoneurons have several anatomical and histological differences compared to SIF motoneurons. Firstly, MIF motoneurons are smaller than SIF motoneurons and INTs (Büttner-Ennever et al., 2001). Secondly, on average, they are contacted by fewer presynaptic buttons originating from non-saccadic premotor areas in contrast to SIF motoneurons and INTs of abducens nucleus (Ugolini et al., 2006; Erichsen et al., 2014). Lastly, SIF motoneurons, as well as INTs, contain various proteins, such as parvalbumin, non-phosphorylated neurofilaments, and perineuronal net (PN, specialized condensed extracellular matrix which surround soma and dendrites of some neurons) markers such as chondroitin sulfate proteoglycan (CSPG) and aggrecan (ACAN), which are all absent in MIF motoneurons (Eberhorn et al., 2005; Horn et al., 2008, 2018; Mayadali et al., 2021). The differential localization and protein expression patterns of MIF and SIF motoneurons have been used to characterize the profile of ion channels and transmitter-related proteins of MIF and SIF motoneurons in monkey (Zeeh et al., 2015; Mayadali et al., 2021). It further served to identify the homologue motoneuron groups in human oculomotor and abducens nuclei (Horn et al., 2008, 2018). The characterization of neurons in the monkey and human abducens nucleus revealed mostly comparable results in terms of localization and anatomical organization of MIF and SIF motoneurons (Horn et al., 2018; Mayadali et al., 2021). However, the overall anatomical characterization of MIF and SIF motoneurons within the human trochlear nucleus has not been reported yet.

Despite clear anatomical and histochemical distinctions found between MIF and SIF motoneurons in various species (Xenopus larvae, cat, monkey), the activity of these neurons is not determined by the eye movement type (Hernandez et al., 2019; Carrero-Rojas et al., 2021; Pastor et al., 2022). Instead, they contribute to several eye movements, albeit with different firing characteristics and therefore yielding differential muscle tension during distinct eye movements (Pastor et al., 2022).

Several key differences in histochemical properties relating to the activity of MIF and SIF motoneurons have been previously revealed in monkey abducens and trochlear nuclei (Mayadali et al., 2021). MIF motoneurons, compared to SIF motoneurons, exhibited reduced immunolabeling of Kv1.1 and Kv3.1 channels, whose co-expression enables fast-firing capabilities of a neuron (Kodama et al., 2020; Mayadali et al., 2021).

Low voltage activated cation channels were another group of ion channels that showed differences in immunolabeling of MIF and SIF motoneurons in monkey trochlear and abducens nuclei. Firstly, HCN1 channel subunit was absent in MIF motoneurons, whereas SIF motoneurons exhibited strong membrane-labeling (Mayadali et al., 2022). Moreover, T-type calcium channel subunit Cav3.1 was prominent in MIF motoneurons, in contrast to only few SIF motoneurons having weak Cav3.1 labeling (Mayadali et al., 2021). The distribution of these channel types in motoneurons points toward their contributions to yield differences in their excitability, spontaneous firing and readiness for post-inhibitory rebound in the firing rate that is fundamental for saccade generation. It is further predicted that high expression of HCN1 in SIF motoneurons would be seen in eye movement system that is more likely to be involved in high-velocity movements, such as horizontal saccades. Preliminary results of this study were presented in the International Brain Research Organization (IBRO) World Congress of Neuroscience 2023 and an abstract was published in IBRO Neuroscience Reports (Mayadali et al., 2023).

## 2 Motivation

The ion channel proteins that showed different labeling patterns for MIF and SIF motoneurons (as well as INTs) in monkey abducens (nVI) and trochlear (nIV) nuclei has not been investigated in human motor nuclei. Therefore, we are first going to establish the anatomical organization and distribution of MIF and SIF motoneurons in the human trochlear (and abducens) nucleus. The main goal of this study is to investigate potential differences in immunolabeling of ion channels with rapid kinematics that are fundamental to serve rapid eye movements, such as saccades. Specific comparisons will be made for voltage-gated potassium channels (Kv1.1 and Kv3.1), and low voltage activated cation channels (HCN) in human trochlear and abducens nuclei. Our *a priori* prediction is that neuronal substrate serving fast dynamics muscle fibers, such as SIF motoneurons, will be equipped more with fast kinematic ion channels, for instance HCN1, while MIF motoneurons will have weaker expression of these channels. These differences will be absent for slow kinematic ion channels, such as HCN4, and for voltage-gated potassium channels, such as Kv channels.

## 3 Materials and methods

### 3.1 Brain tissue

Brains extracted from 3 human cases (H1, H2, H3) with post-mortem delays up to 27 h were fixed in 4% paraformaldehyde solution in 0.1M phosphate buffer (pH 7.4) for 2–7 days. The age of the donors ranged from 69 to 80 years, and there was no known history of neurological disease. The brainstems were cut into 1–2 cm thick tissue blocks, which were then embedded in paraffin for sectioning in the transverse plane. 5–10 μm thick paraffin sections were then processed with peroxidase based immunohistochemistry methods identical to previously described protocol in monkey abducens and trochlear nuclei (Mayadali et al., 2021). Information on primary antibodies specifically used in this study, are summarized in Table 1.

**TABLE 1:** Summary of primary antibodies and dilutions for the immunolabeling.

Antibody	Host	Antigen	Manufacturer	Antibody registry number (RRID)	Dilution
ACAN	Mouse/monoclonal	Aggrecan	Acris Antibodies GmbH, 32052 Herford, Germany	AB_972582	1:75
ChAT	Goat/polyclonal	Choline acetyltransferase	Chemicon, Temecula, CA, USA	AB_2079751	1:50
Kv1.1	Rabbit/polyclonal	Voltage-gated potassium channel 1.1	Alomone Labs Jerusalem BioPark (JBP)	AB_2040144	1:500
Kv3.1b	Rabbit/polyclonal	Voltage-gated potassium channel 3.1b	Weiser et al., 1995	Härtig et al., 1999 (AB_2040166)	1:5000
HCN1	Rabbit/polyclonal	Hyperpolarization-activated cyclic nucleotide-gated channel 1	Thermo Fischer Scientific, MA, USA	AB_2735891	1:400
HCN2	Rabbit/polyclonal	Hyperpolarization-activated cyclic nucleotide-gated channel 2	Thermo Fischer Scientific, MA, USA	AB_2735892	1:1500
HCN4	Guinea pig/polyclonal	Hyperpolarization-activated cyclic nucleotide-gated channel 4	Alomone Labs Jerusalem BioPark (JBP)	AB_2340957	1:200

### 3.2 Image acquisition/analysis

Paraffin sections of human trochlear nucleus (nIV) stained for choline acetyltransferase (ChAT) and aggrecan (ACAN), as well as for ion channel proteins, were imaged using a slide scanner (Mirax MIDI, Zeiss) equipped with a plan apochromate objective (×20). The digitized images were viewed and captured on a computer with the free software Case Viewer (3DHistech; v.2.2) and Slide Viewer (3DHistech; version 2.6). Corresponding detailed views of equally arranged and magnified images of adjacent sections were analyzed on the computer screen. The same neurons were identified by their location with the help of anatomical landmarks, such as blood vessels. For plotting of the motoneuron types, the images of ChAT/ACAN stained sections were taken as template using drawing software (Coreldraw 11.0; COREL). This software was also used to arrange and label the figure plates.

### 3.3 Description of measurements and statistical analysis

Motoneuron populations were characterized in terms of their size by measuring their average somatic diameters [(d_max_+d_min_)/2] with the software ImageJ. These average somatic diameter measurements were used as an indicator of neuronal size in the analyses seen in Figures 3, 4. Statistical significance of the differences observed in average diameter for MIF, weak-PN and SIF motoneurons was examined within each case (Figure 3) by two-tailed student’s *t*-test for two independent samples. The mean values of the individual populations were compared with each other for the analysis of statistically significant difference.

MIF, weak-PN and SIF motoneurons in anatomically identified abducens and trochlear nuclei were classified as follows: 1-MIF motoneurons are ChAT-positive neurons with no PN fragments enveloping or in contact with their cell membrane; 2- Weak-PN motoneurons are ChAT-positive neurons with some PN fragments in direct contact with cell membrane without enveloping the soma; SIF motoneurons are ChAT-positive neurons with strong and dense continuous PN envelopment of soma and proximal dendrites.

Cell size profiles of trochlear motoneuron types were analyzed in all three cases with excel functions (Microsoft Office v16). Motoneurons, which were manually selected and separated into MIF, SIF and weak-PN groups were listed according to their sizes. This was done by excel’s “frequency” function. Average diameter measurements of each motoneuron population were binned into 16 groups covering 4 μm increments from 0 to 60 μm. The number of neurons fitting into these bins were averaged across total number of motoneurons within each case. The results were plotted demonstrating the possibility of encountering a particular type of neuron at a particular size (Figure 4).

## 4 Results

### 4.1 Localization of different motoneuron types in the human trochlear nucleus

**FIGURE 1:**
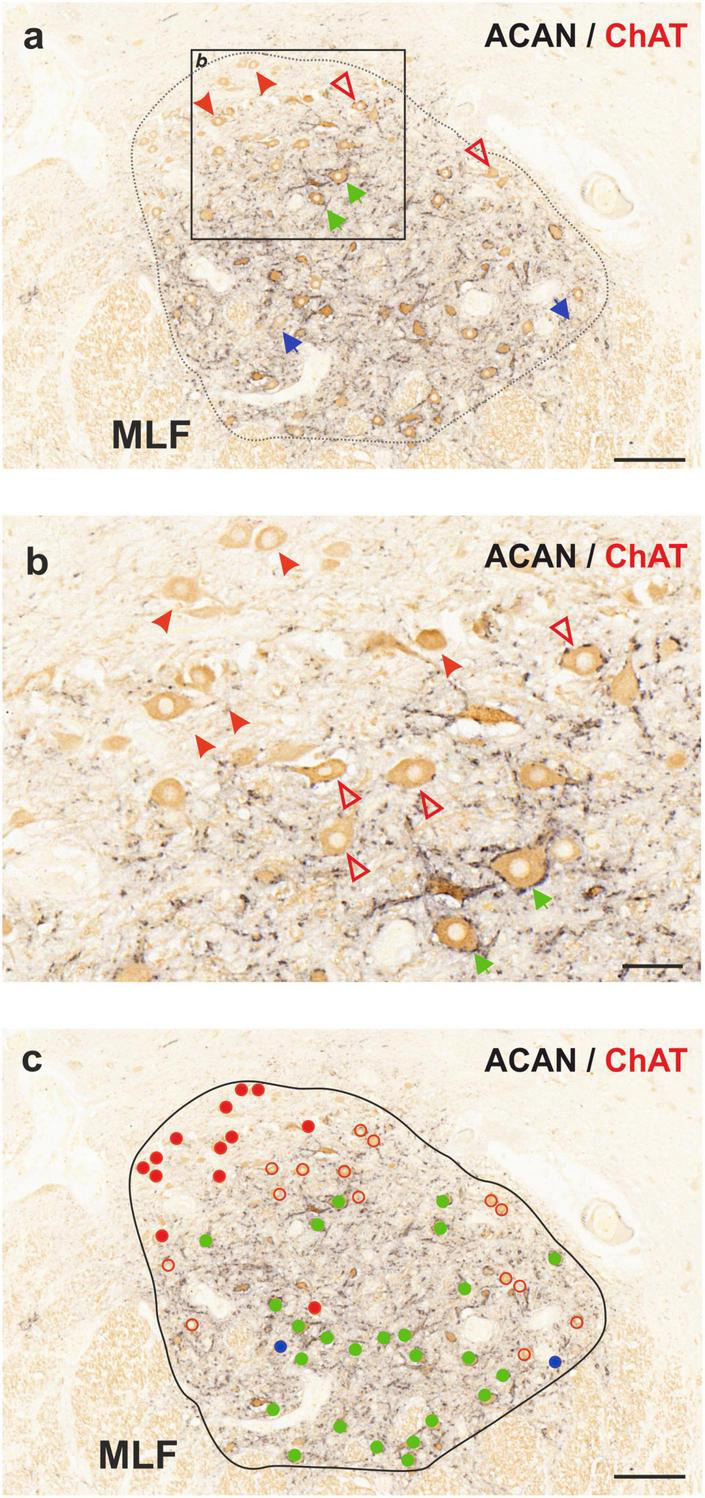
Immunoperoxidase-based histochemical classification of motoneurons in human trochlear nucleus (nIV). **(a)** Combined peroxidase labeling of choline acetyltransferase (ChAT, brown) and perineuronal net (PN) marker aggrecan (ACAN, black) reveals motoneurons of singly-innervated muscle fibers (SIF, green arrows), motoneurons of multiply-innervated muscle fibers (MIF, red arrowheads) and a third motoneuron group with intermediary characteristics (weak-PN, red open arrowheads). ChAT-negative neurons with ACAN-positive PN represent internuclear neurons (INT) indicated by blue arrows. The box indicates the area illustrated at higher magnification in **(b)**. **(b)** Close-up demonstrating MIF motoneurons lacking PN ensheathment (red arrowheads), SIF motoneurons with strong PNs (green arrows) and motoneurons with weak PN expression (red open arrowheads). **(c)** Distribution of three sub-populations of motoneurons and internuclear neurons in the human trochlear nucleus. MIF motoneurons are denoted with red filled-circles, SIF motoneurons with green filled-circles and the weak-PN motoneuron group is denoted by red empty circles, INTs by blue filled-circles. MLF, medial longitudinal fascicle. Scale bar represents 200 μm in **(a,c)** and 50 μm in **(b)**.

**FIGURE 2:**
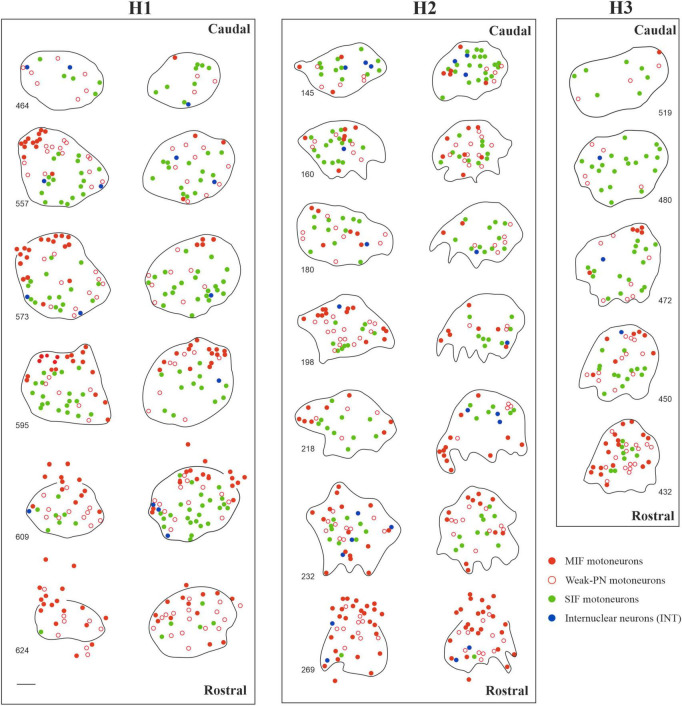
Localization of motoneuron types in human trochlear nucleus in three cases (H1, H2 and H3). Reconstructions of trochlear nucleus were depicted bilaterally for H1 and H2, and from the right side for H3 in multiple digitalized sections at different transverse planes as displayed in the caudo-rostral axis. MIF motoneurons (red dots) were often found lying in the dorsal and rostral portion of trochlear nuclei. In one case (H1, left), MIF motoneurons formed a dorsal-cap similar to the trochlear nucleus of macaque monkey. In the other cases, MIF motoneurons were distributed more diffusely but more clustered at the rostral planes. SIF motoneurons (green dots) were found all over the nucleus, with slight prevalence of ventral and lateral placement. Lastly, acetylcholine-negative putative internuclear neurons with strong PN ensheathment (blue dots) were sparsely found within the nucleus. Scale bar indicates 200 μm.

In order to examine the anatomical organization of the human trochlear nucleus motoneurons, series of transverse sections were simultaneously immunostained with ACAN (a key component of the PN) and choline acetyltransferase (ChAT) antibodies, in a similar fashion to the work on abducens nucleus (Horn et al., 2018; Figure 1a). Analysis of ACAN/ChAT immunohistochemistry performed on all three human cases revealed the localization of SIF motoneurons with strong PN ensheathment (Figures 1a, b; green arrows; Figure 1c; green filled circles) and MIF motoneurons lacking PNs (Figures 1a, b; arrowheads; Figure 1c; red filled circles). Remarkably, a considerable number of motoneurons was only weakly labeled with ACAN antibody, and therefore these were ruled out as MIF motoneurons (Figures 1a, b; red open arrowheads). Due to its consistent occurrence in human tissue, we classified this “weak-PN” group as a third group of motoneurons, lying between MIF and SIF motoneurons in terms of PN immunolabeling for further analysis (Figure 1b; red open arrowheads). In order to determine the localization of all motor neuron populations in trochlear nucleus, reconstructions were created on digitally captured images of stained sections, in which the motor neuron types were drawn as dots with different colors (Figure 1c). Findings were evaluated and plotted for the entire trochlear nucleus at different planes caudo-rostrally for three human cases (Figure 2). In all cases, MIF motoneurons were found more concentrated toward the dorsal border of the trochlear nucleus, and outnumbered SIF motoneurons toward the rostral end along the rostro-caudal axis (Figure 2, H1, H2, H3; red dots), comparable to monkey trochlear nucleus (Mayadali et al., 2021). In one case, motoneurons with weak PNs lay mainly between MIF and SIF motoneurons (Figure 2, H1; red circles). This distribution pattern was less obvious in other cases, where motoneurons with weak PNs did not form an intermediate group between MIF and SIF motoneurons in terms of localization but were found scattered within trochlear nucleus (Figure 2, H2; H3 red circles). MIF motoneurons were found more scattered within trochlear nucleus at caudal levels, but more concentrated at the rostral border (Figure 2, H2, H3; red dots). Lastly, according to abducens nucleus non-cholinergic neurons with strong perineuronal nets in trochlear nucleus were considered as internuclear neurons (INTs) that were found in small number in monkey projecting to prepositus and/or abducens nucleus (see Langer et al., 1986; Belknap and McCrea, 1988). Here putative INTs were found sparsely in trochlear nucleus in all cases (Figure 1a; blue arrows; Figure 1c; blue dots) (Figure 2; blue dots) (Table 2).

**TABLE 2:** Summary of motoneuron type distributions in human trochlear nucleus.

	SIF MN	MIF MN	Weak-PN	INT
H1	35.22%	32.89%	28.49%	3.41%
H2	31.61%	33.46%	30.14%	4.80%
H3	41.51%	26.42%	29.43%	2.64%
Total	34.62%	32.29%	29.24%	3.85%

The percentages of various motoneuron types and internuclear neurons forming the trochlear nuclei of three cases shown. H1-3 indicate the case numbers. SIF MN, motoneurons of singly-innervated fibers. MIF MN, motoneurons of multiply-innervated fibers. Weak-PN, motoneuron population with weak perineuronal nets. INT, internuclear neurons. Total MNs, the sum of all motoneurons in three cases.

### 4.2 Characterization of motoneurons in the human trochlear nucleus

The neuron types in trochlear nucleus of three human cases were quantified (H1: *n* = 1,025; H2: *n* = 813; H3: *n* = 265) (Table 2). The largest population was formed by SIF motoneurons (H1: 35.22%; H2: 31.61%; H3: 41.51%) and followed by MIF motoneurons (H1: 32.89%; H2: 33.46%; H3: 26.42%) and weak-PN motoneurons (H1: 28.49%; H2: 30.14%; H3: 29.43%). Only few non-cholinergic putative INTs were present in trochlear nucleus (H1: 3.41%; H2: 4.80%; H3: 2.64%). Of all trochlear neurons quantified (*n* = 2,103) over all cases, 34.62% were SIF (*n* = 728), 32.29% were MIF motoneurons (*n* = 679), 29.24% (*n* = 615) were motoneurons with weak PNs and 3.85% were INTs (*n* = 81) (Table 2).

Previous work in monkey has shown that MIF motoneurons are smaller in size compared to SIF motoneurons (Büttner-Ennever et al., 2001; Eberhorn et al., 2005). Therefore, the existence of a putative intermediate third group of motoneurons (weak-PN) was further scrutinized morphologically. In order to achieve this, motoneuron populations were characterized in terms of their average diameters. Average MIF motoneuron diameter was 24.54 μm (*n* = 104; STD = 3.78) for case H1, 26.83 μm (*n* = 95, STD = 4.15) for case H2 and 32.37 μm (*n* = 26, STD = 3.52) for case H3. Average diameter of weak-PN motoneurons was 28.79 (*n* = 92, STD = 4.28) for H1, 30.28 (*n* = 87, STD = 4.15) for H2 and 37.40 (*n* = 39, STD = 5.13) for H3. Finally, average diameter for SIF motoneurons was 30.75 (*n* = 139, STD = 5.02) for H1, 35.78 (*n* = 132, STD = 5.36) for H2, and 45.16 (*n* = 54, STD = 6.88) for H3.

**FIGURE 3:**
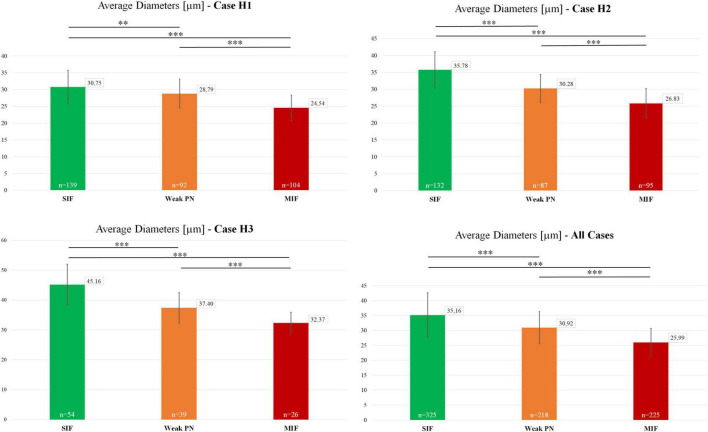
Characterization and size-comparison of motoneuron types in the human trochlear nucleus. Mean diameters of motoneurons identified as SIF (green), MIF (red) and weak-PN group (orange) were calculated as (d_max_+d_min_)/2. Number of measured neurons are given within the graph bars. Average diameters were numerically provided in the small boxes at the top-right corner of the graph bars. Lastly, statistical analysis (two-tailed *t*-test for two independent means), as shown above the bars, indicated significant differences between the mean diameters of these three motoneuron populations (**p < 0.01, ***p < 0.001).

For the H1 case, the average diameters of SIF and weak-PN motoneurons were significantly different (*p* < 0.01; *t*-test; Figure 3). The difference between MIF and weak-PN motoneuron size, as well as between SIF and MIF motoneurons, was also significantly different (*p* < 0.001; *t*-test; Figure 3). The significant difference between the mean diameter of three distinct populations for H1 was confirmed with one-way ANOVA (f-ratio: 58.47; *p* < 0.00001). For the H2 and H3 cases, all three motoneuron populations were also significantly different in size when compared in pairs (*p* < 0.001; *t*-test; Figure 3). One-way ANOVA analysis for H2 and H3 revealed statistically significant differences between average diameters of the three motoneuron populations (f-ratio: 122.02; *p* < 0.00001 for H2; f-ratio: 47.72; *p* < 0.00001 for H3, respectively). Analysis of all SIF (*n* = 325), MIF (*n* = 225) and weak-PN (*n* = 218) quantified motoneurons revealed that the mean diameters were significantly different between each of these populations as shown in pairs (*p* < 0.00001; *t*-test; Figure 3) and with one-way ANOVA test (f-ratio: 98.97; *p* < 0.00001).

**FIGURE 4:**
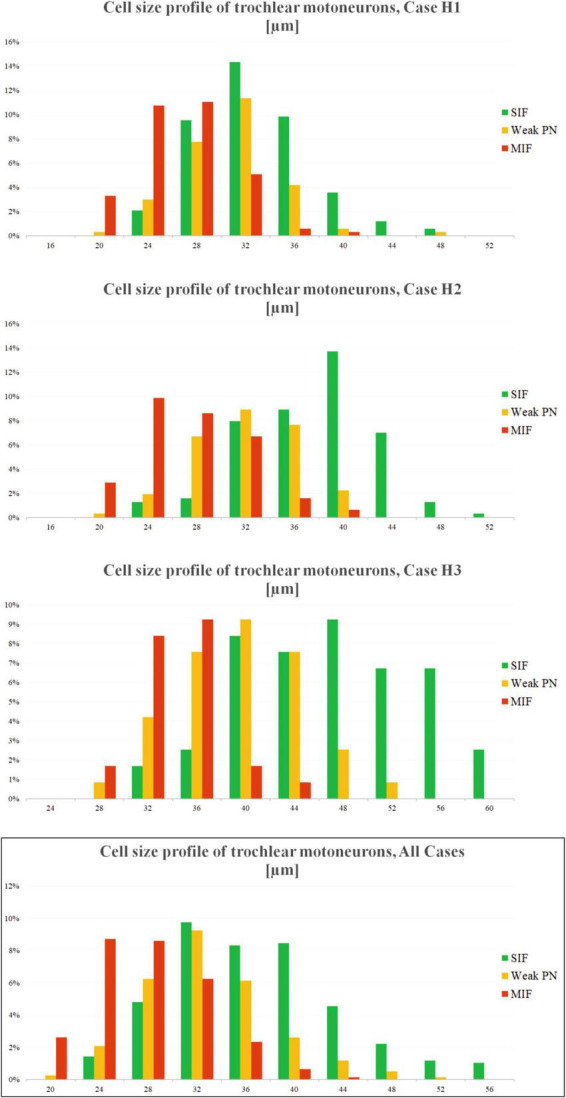
Distribution of motoneuron types across the neuronal diameters for three cases (H1, H2 and H3). *X*-axis of the plot denotes the neuronal diameter, *Y*-axis denotes the chance percentage of encountering a certain type of neuron at that size. The bar height, therefore, indicates how many percent of the given motoneurons (for all three types) are encountered at that diameter. Analysis yields three overlapping (normal) distributions with most MIF motoneurons (red) found toward the smallest diameters, and SIF motoneurons (green) found toward the biggest diameters. Motoneuron population with weak PNs (orange) was found most prominent between these two populations. Additionally, chance of finding a MIF motoneuron at bigger sizes, or SIF motoneuron at smaller sizes, are quite low.

In order to confirm further the characterization of the three motoneuron populations identified with varying PN ensheathment, we measured each motoneuron according to its size, identified what type of neuron it is at a certain diameter, and plotted the frequency of occurrence of that particular motoneuron type at the given size (Figure 4). The normal/bell-curve distributions of motoneuron pools according to their sizes were visible for each case (Figure 4). Additionally, this visualization in all three cases revealed that MIF motoneuron population dominates the smallest motoneuron range. For instance, MIF motoneuron population was the most prominent in the 20–28 μm diameter range (Figure 4, H1, H2; red bars). On the other hand, SIF motoneurons with strong PNs dominate the right-hand side of the motoneuron distribution (Figure 4; green bars) as they consisted of the most prominent group at 36 μm and bigger. As expected, weak-PN motoneuron population peaked in the middle range of the overall distribution (Figure 4; orange bars). For instance, 41% of weak-PN motoneuron population was found at ∼32 μ diameter and was the most prominent population in the H1 case at this size, consisting more than 8% out of all quantified motoneurons (Figure 4, H1; orange bars).

### 4.3 Voltage-gated potassium (Kv) channels in the MIF and SIF motoneurons of the human trochlear and abducens nuclei

**FIGURE 5:**
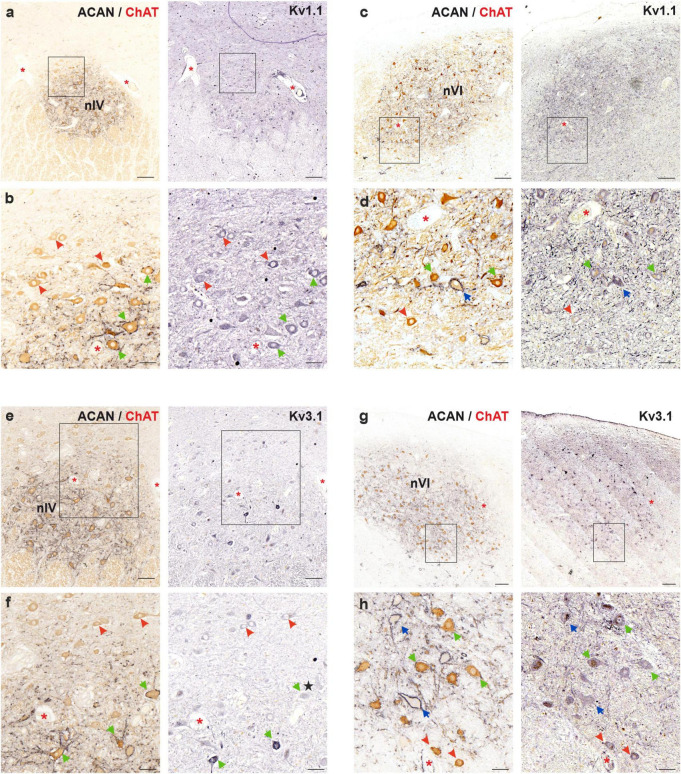
Immunoperoxidase labeling of Kv1.1 (top) and Kv3.1b (bottom) proteins in the trochlear (nIV, left) and abducens nucleus (nVI, right). **(a)** Consecutive 5 μm thick coronal paraffin sections through trochlear nucleus stained for Kv1.1. The box indicates the area illustrated at higher magnification in **(b)**. **(b)** Close-up of Kv1.1 labeling in motoneurons. Note the similar immunolabeling density in MIF (red arrowheads) and SIF (green arrows) motoneurons with little variation. **(c)** Consecutive 5 μm thick coronal paraffin sections through abducens nucleus stained for Kv1.1. The box indicates the area illustrated at higher magnification in **(d)**. **(d)** Close-up of Kv1.1 labeling in motoneurons. Note the slight variation in Kv1.1 immunolabeling density between neuron types: MIF (red arrowheads) and SIF (green arrows) motoneurons, as well as internuclear neurons (INTs, blue arrows). **(e)** Consecutive 5 μm thick coronal paraffin sections through trochlear nucleus stained for Kv3.1b. The box indicates the area illustrated at higher magnification in **(f)**. **(f)** Close-up of Kv3.1b labeling in motoneurons. Note the varied levels of immunolabeling density within SIF motoneurons (green arrows). Some SIF motoneurons shows stronger Kv3.1b immunolabeling than MIF motoneurons (red arrowheads) but several SIF motoneurons express similar level of immunolabeling (star). **(g)** Consecutive 5 μm thick coronal paraffin sections through abducens nucleus stained for Kv3.1b. The box indicates the area illustrated at higher magnification in **(h)**. **(h)** Close-up of Kv3.1b labeling in motoneurons and INTs (blue arrows) in abducens nucleus. Note similar levels of Kv3.1b immunolabeling in all three neuron types. Red asterisks indicate blood vessels as landmarks for orientation in neighboring sections. Scale bars indicate 200 μm in **(a,c,e)**, and g; 50 μm in **(b,d,f,h)**.

5 μm thick consecutive frontal paraffin sections of human trochlear and abducens nucleus were immunostained with Kv1.1, ACAN/ChAT and Kv3.1b antibodies, respectively, and were analyzed qualitatively for immunolabeling density (Figure 5). Immunostaining against Kv1.1 (Figures 5a, c) revealed no consistent difference in immunolabeling density between MIF (red arrowheads) and SIF (green arrows) motoneurons (Figures 5b, d). Immunolabeling density did not vary within the trochlear nucleus, including MIF motoneurons, contrary to the observations in monkey motor nuclei (Figure 5a; Mayadali et al., 2021). On the other hand, Kv3.1b immunolabeling yielded mixed results in trochlear nucleus (Figures 5e, f), where both MIF and SIF motoneurons were immunolabeled, without consistent qualitative difference (Figures 5e, f). Some SIF motoneurons expressed a notably stronger immunolabeling for Kv3.1b than MIF motoneurons (Figure 5f; green arrows), but many SIF motoneurons were weakly-labeled similar to MIF motoneurons (Figure 5f; black star).

In abducens nucleus, Kv1.1 immunolabeling did not strongly differ between MIF and SIF motoneurons (Figures 5c, d), however, it showed variability within all three populations. Similar results were observed for Kv3.1b antibody labeling (Figures 5g, h) as there were no consistent differences between MIF and SIF motoneurons. As for Kv1.1, Kv3.1b labeling density varied within the abducens nucleus in all three populations (Figure 5g).

### 4.4 HCN channels in human trochlear and abducens nuclei

**FIGURE 6:**
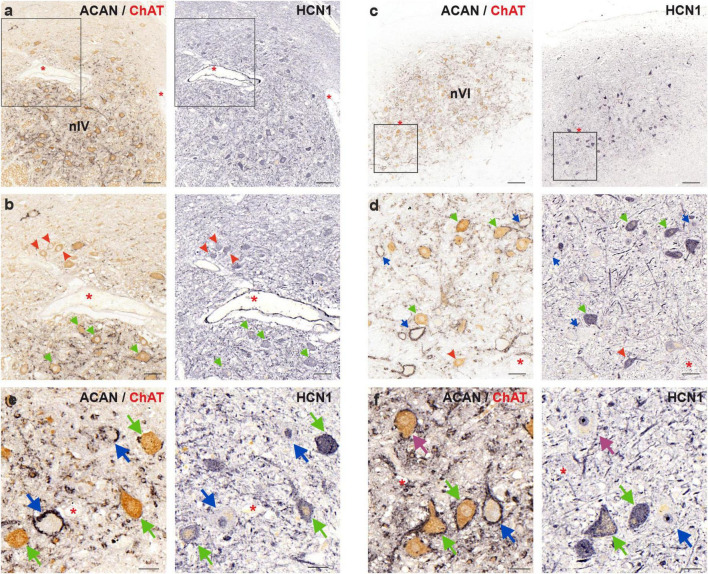
Immunoperoxidase labeling of HCN1 protein in the human trochlear (nIV, left) and abducens nucleus (nVI, right). **(a)** Consecutive 5 μm thick coronal paraffin sections through trochlear nucleus stained for HCN1. The box indicates the area illustrated at higher magnification in **(b)**. **(b)** Close-up of HCN1 labeling in motoneurons. Note the similar immunolabeling density in MIF (red arrowheads) and SIF (green arrows) motoneurons. **(c)** Consecutive 5 μm thick coronal paraffin sections through abducens nucleus stained for HCN1. The box indicates the area illustrated at higher magnification in **(d)**. **(d)** Close-up of HCN1 labeling in motoneurons and internuclear neurons (INT, blue arrows) demonstrating similar HCN1 staining of SIF and MIF motoneurons. **(e,f)** Faint HCN1 expression in INTs compared to strong labeling of SIF motoneurons in trochlear **(e)** and abducens **(f)** nuclei. A SIF motoneuron with negative somatic HCN1 immunolabeling is indicated with a purple arrow. Red asterisks indicate blood vessels as landmarks for orientation in neighboring sections. Scale bars indicate 200 μm in **(b)**, 100 μm in **(a)**, 50 μm in **(b,d)**, and 30 μm in **(e,f)**.

**FIGURE 7:**
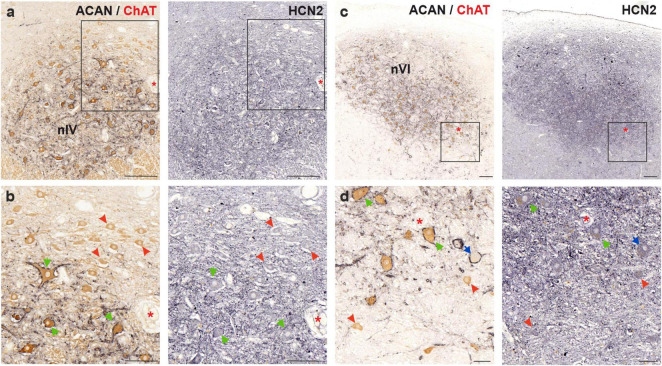
Immunoperoxidase labeling of HCN2 protein in the human trochlear (nIV, left) and abducens nucleus (nVI, right). **(a)** Consecutive 5 μm thick coronal paraffin sections through trochlear nucleus stained for HCN2. The box indicates the area illustrated at higher magnification in **(b)**. **(b)** Close-up of HCN2 labeling in motoneurons. Note the stronger punctate immunolabeling in SIF (green arrows) compared to MIF (red arrowhead) motoneurons. **(c)** Consecutive 5 μm thick coronal paraffin sections through abducens nucleus stained for HCN2. Note the intense neuropil labeling within the borders of the abducens nucleus. The box indicates the area illustrated at higher magnification in **(d)**. **(d)** Close-up of abducens motoneurons and an INT (blue arrow) demonstrating similar punctate immunolabeling between MIF (red arrowhead), SIF (green arrows) and INTs. Red asterisks indicate blood vessels as landmarks for orientation in neighboring sections. Scale bars: 200 μm in **(a,c)** 100 μm in **(b)** and 50 μm in **(d)**.

**FIGURE 8:**
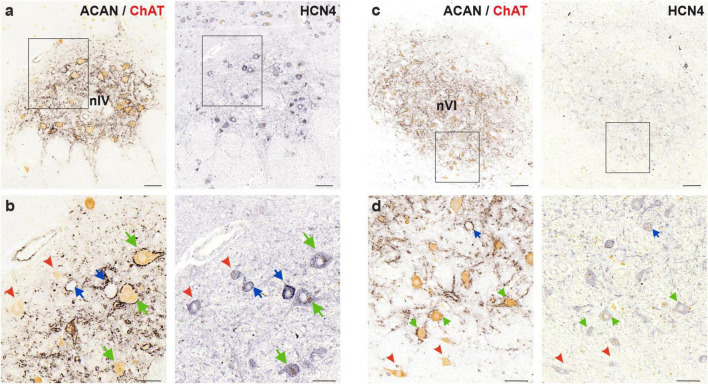
Immunoperoxidase labeling of HCN4 protein in the human trochlear (nIV, left) and abducens nucleus (nVI, right). **(a)** Consecutive 5 μm thick coronal paraffin sections through trochlear nucleus stained for HCN4. The box indicates the area illustrated at higher magnification in **(b)**. **(b)** Close-up of HCN4 labeling in motoneurons and putative internuclear neurons (INTs, blue arrows). Note the comparable levels of immunolabeling density in MIF (red arrowheads) and SIF (green arrows) motoneurons, however, slightly higher intensity labeling in INTs (blue arrows). **(c)** Consecutive 5 μm thick coronal paraffin sections through abducens nucleus stained for HCN4. The box indicates the area illustrated at higher magnification in **(d)**. **(d)** Close-up of HCN1 labeling in motoneurons and internuclear neurons (INT, blue arrows) demonstrating similar HCN1 staining of SIF and MIF motoneurons as well as INTs. Scale bars indicate 200 μm in **(c)**, 100 μm in **(a)**, 50 μm in **(b,d)**.

5 μm thick consecutive frontal paraffin sections of human abducens and trochlear nuclei were immunostained with HCN1, ACAN/ChAT and antibodies against HCN2/HCN4, respectively, in order to investigate HCN channel subunit distribution and expression in MIF and SIF motoneurons (Figures 6–8).

#### 4.4.1 HCN1 channel

In the human trochlear nucleus, both SIF and MIF motoneurons were immunolabeled with HCN1 antibody, without a clear difference in labeling density (Figures 6a, b; green and red arrows, respectively). On the other hand, in the abducens nucleus, MIF motoneurons (Figures 6c, d; red arrowheads) showed a slightly weaker immunolabeling for HCN1 compared to SIF motoneurons (Figure 6d; green arrows). In both groups, HCN1 immunolabeling appeared on the somatic and dendritic membrane as well as within the somata of the motoneurons.

Analysis in the human abducens nucleus revealed that most SIF motoneurons expressed strong HCN1 immunolabeling (Figures 6d, f; green arrows). However, INTs were unlabeled with HCN1, except for few weakly labeled neurons (Figures 6d, f; blue arrows). Moreover, close examination of the neurons that have the same histochemical signature (PN-positive ChAT-negative) within trochlear nucleus (therefore considered as putative internuclear neurons) revealed that these neurons likewise lacked HCN1 immunolabeling (Figure 6e; blue arrows). Interestingly, few SIF motoneurons with strong PN expression but weak or absent HCN1 expression were encountered (Figure 6f; purple arrow).

#### 4.4.2 HCN2 channel

HCN2 subunit immunolabeling in human trochlear and abducens nuclei revealed strong neuropil labeling that distinguished their anatomical boundaries (Figures 7a, c). As for the staining of motoneurons, punctate labeling on the membrane with additional somatic labeling was observed. In the trochlear nucleus, punctate labeling intensity on MIF motoneurons was weaker compared to SIF motoneurons (Figures 7a, b; red arrowheads and green arrows, respectively). Additionally, a general observation about a correlation between HCN2 punctate immunolabeling density and PN density could be made.

In the abducens nucleus, HCN2 antibody labeled the neuronal membranes, as well as neuropil within the nucleus, presumably labeling dendritic processes (Figures 7c, d). Both MIF and SIF motoneuron somata were labeled by punctate HCN2 staining (Figure 7d; red arrowheads and green arrows, respectively). However, the difference in immunolabeling density between MIF and SIF motoneurons in abducens nucleus was not clear due to the neuropil labeling (Figures 7c, d). Abducens INTs were also labeled by HCN2 antibody, in a similar fashion, with puncta across their somatic membranes (Figure 7d; blue arrow).

#### 4.4.3 HCN4 channel

Lastly, the slowest-activated member of the HCN channel family, HCN4, was investigated in MIF and SIF motoneurons of the human abducens and trochlear nuclei (Figure 8). A moderate membrane- and somatic-immunolabeling pattern was observed in both MIF (red arrowheads) and SIF motoneurons (green arrows), with no obvious qualitative differences between these neuron types within each nucleus (Figures 8b, d).

To investigate whether the HCN channel expression in the INTs skewed toward the slower-activated members of the HCN channels, HCN4 expression was examined in putative INTs in both abducens and trochlear nuclei. In trochlear nucleus, several (putative) INTs showed stronger immunoreactivity for HCN4 subunit (Figure 8b; blue arrows). However, this phenomenon was not observed in abducens nucleus (Figure 8d). In comparison to SIF motoneurons, INTs were likewise immunopositive for HCN4 subunit antibody with similar immunolabeling density (Figure 8d; green and blue arrows, respectively).

## 5 Discussion

This study investigated ion channel profiles of extraocular motoneurons and internuclear neurons in human abducens and trochlear nuclei. Another focus of this investigation involved characterization of motoneurons of multiply-innervated muscle fibers (MIF), and of singly-innervated muscle fibers (SIF) in the human trochlear nucleus. A qualitative examination suggested the existence of an intermediary group in terms of the perineuronal net (PN) ensheathment density, and this hypothesis was confirmed to have significance due to the correlation between PN density and neuronal size distribution. On the other hand, the analysis of ion channel profiles of MIF and SIF motoneurons often yielded no clear differences in terms of immunolabeling density. One striking finding was the lack of immunolabeling with HCN1 antibody in abducens (and trochlear) internuclear neurons, compared to strong labeling of most SIF motoneurons.

### 5.1 Localization of MIF and SIF motoneurons in human trochlear nucleus

The organization of the trochlear nucleus seemed roughly similar in monkey and human in terms of MIF and SIF motoneuron localization. MIF motoneurons, identified with positive ChAT and negative PN immunoreactivity, were usually concentrated in the dorsal and anterior portion of the trochlear nucleus. In monkey trochlear nucleus, MIF motoneurons form a dorsal cap with clear boundaries separating them from SIF motoneurons and are found primarily at more rostral levels as seen here in human (Büttner-Ennever et al., 2001; Mayadali et al., 2021).

As in monkey trochlear nucleus, MIF motoneurons of human trochlear nucleus were, on average, smaller than SIF motoneurons as demonstrated in Figures 1, 3, 4, which confirmed the earlier preliminary observations on Nissl-stained sections (Ardeleanu, 2007).

### 5.2 Assumption of a “third” motoneuron population with weak perineuronal nets

Although not looked for, the striking and consistent encounter of cholinergic neurons with only weak PNs was indicative of a distinct group of motoneurons differing from classically defined MIF and SIF motoneurons. The presupposition of a “third” intermediary group placed between characteristically MIF and SIF motoneurons was based on the qualitative observations and was further investigated for the correlation to neuronal size. The statistical analysis demonstrating significant differences in the mean diameters to both MIF and SIF motoneurons established the legitimacy of our qualitative observation (Figure 3). Furthermore, the probability of occurrence for all three populations at varying diameters showed a virtually normal, bell-curve distribution, with each of the three populations peaking at different diameters (Figure 4). These results confirmed the potential weak-PN motoneuron group as a third and intermediate population to histochemically identified MIF and SIF motoneurons, as qualitative characterization was supported by statistical analysis.

In theory, this intermediary weak-PN group may represent motoneurons targeting the MIFs of the orbital layer, which show specific properties (Davidowitz et al., 1996). As global MIFs, those of the orbital layer are innervated by multiple typical en grappe endplates but they have additional central en plaque endings (Davidowitz et al., 1977; Lucas et al., 2018). Accordingly, corresponding contraction dynamics were observed in central and distal portions upon electrical stimulation of the innervating axons (Jakoby et al., 1989).

Tract-tracer injections into the orbital layer of lateral rectus muscle in cat did not reveal an anatomically distinct population of motoneurons compared with complete muscle belly injections (Bohlen et al., 2017) as seen for global SIF and MIF motoneurons (Büttner-Ennever et al., 2001). Therefore, in theory, it is possible that in human orbital MIF motoneurons are intermingled with SIF motoneurons and exhibit distinct physiological characteristics, which are reflected by weak perineuronal nets. The smaller neurons within the weak PNs could correspond to the orbital MIF motoneuron characteristics. In our analysis, SIF and weak-PN motoneuron populations together constitute 60–70% of all motoneurons (see Table 2). This number is still lower than the estimated SIF motoneuron ratio in both orbital and global layers from muscle fiber number [80:20 ratio of SIF:MIF, (Oh et al., 2001; Spencer and Porter, 2006)]. On the other hand, we do not know the exact sizes of motor units for all motoneuron types including the possibility of polyneuronal innervation of MIFs (Goldberg et al., 1998). However, our research cannot conclusively answer this question, as our methodology used in this study is limited to histochemistry of brainstem sections. The average numbers of 26–32% MIF motoneurons versus 60–70% SIF motoneurons also differs from previous studies from thick sections of monkey trochlear nucleus, where a lower percentage of 19% MIF motoneurons was found (Eberhorn et al., 2005). Since our analysis revealed that the proportion of MIF and SIF motoneurons is not equal throughout the whole extent of the trochlear nucleus, our data may represent an overestimation of MIF motoneurons due to the analysis of a limited number of thin paraffin sections.

The assumption of a “third” intermediary motoneuron population, however, must not be treated squarely as suggestion of a third separate functional motoneuron subpopulation, as we can provide no evidence supporting this. In this regard, it is indeed possible to have more functionally/anatomically relevant number of subpopulations with slightly different histochemical characteristics. Thereby this third group, as we prefer to suggest so far, is a sign of a variety, or a range of biophysiological spectrum within the SIF motoneuron population. This is also seen at the level of muscle fibers, where the expression of different combinations of 11 myosin heavy chain isoforms define the contraction properties and dynamics of a given muscle fiber (Hoh, 2021). Based on mitochondrial content and expression of different mitochondrial and sarcoplasmic enzymes, three types of SIFs have been identified: pale, intermediate and red muscle fibers, which may be related to certain range of contraction properties (Wasicky et al., 2000; Kjellgren et al., 2003; Spencer and Porter, 2006). The muscle fiber content could, in turn, affect the neuronal phenotype to develop with varied characteristics (Hernández et al., 2017a,b). It is also quite possible to interpret this data as the traditionally defined MIF and SIF motoneurons simply belong to the opposite ends of a spectrum of motoneurons in terms of their size and their PN content (Horn and Straka, 2021). If we had chosen to split this “weak-PN” population further into two subgroups in terms of relative PN ensheathment density, it is very plausible to assume that the size-PN density correlation would still hold true. Therefore, our arbitrary treatment of the motoneurons with weak PNs as a separate group should be only considered as a method of establishing a correlation between the observable qualities such as size and PN density.

### 5.3 Biophysiological implications of the varied perineuronal net expression

As extensively discussed in our previous work (Horn et al., 2018; Mayadali et al., 2021, 2022), PN-ensheathment density has direct and indirect effects on firing characteristics of neurons through (co)regulating transmitter and ion channel content of neuronal membranes, as well as affecting calcium-binding protein expression of neurons. In this context, the variety of PN expression in human ocular motonuclei could be interpreted as a contribution to a wide range of variety in biophysiological features of motoneurons to generate complex eye movements. This variety is accompanied by cell-size, which can imply differentiation in characteristics on its own (Henneman, 1957). However, it is known that PN density affect biophysiological properties, such as high-firing rate, via co-regulating ion channel subunits related to high-firing capacity (i.e., Kv1.1, Kv3.1) (Härtig et al., 1999; Favuzzi et al., 2017). Interestingly, we did not observe a clear-cut labeling density difference neither between these three putative subpopulations, nor at the MIF-SIF motoneuron level. Therefore, within the scope of this study, it is difficult to assign a strong distinction in firing characteristics of these motoneuron types based on the histochemical staining of Kv channels.

### 5.4 Comparison to monkey tissue

#### 5.4.1 Lack of weak-PN motoneuron subpopulation in monkey abducens and trochlear nuclei

First significant difference observed between monkey and human motoneurons were the existence of a weak-PN motoneuron population as seen in the human trochlear nucleus. Meanwhile, in the monkey abducens and trochlear nuclei, SIF motoneurons presented with a uniform level of PN immunolabeling to such a degree that abducens INT neurons could be reliably discerned from SIF motoneurons by their comparatively stronger PN ensheathment (Mayadali et al., 2021). In human abducens nucleus, INTs could be discerned by their consistent and prominent PN ensheathment contrary to motoneurons, which varied in immunolabeling intensity (Horn et al., 2018). An argument could be made that the age of the human donors in comparison to the relative age of the monkey could play a role in a hypothetical loss of PN integrity, as PN envelopment density could be altered by aging in people, as reported in several neurodegenerative conditions as well as other species (Sugitani et al., 2021; Dong et al., 2023). So far, our unreported research contradicts this, as we have seen the existence of the motoneurons with weak PNs in tissues from donors with varying ages, or with neurodegenerative conditions such as ALS or anti-GAD syndrome associated opsoclonus.

#### 5.4.2 Variable immunolabeling of ion channel proteins in MIF and SIF motoneurons of human trochlear and abducens nuclei

In the monkey abducens and trochlear nuclei, significant differences in immunolabeling density were found between MIF and SIF motoneurons with respect to Kv1.1, Kv3.1, HCN1 and Cav3.1 channels (Mayadali et al., 2021, 2022). Despite the fact that these channels generally exhibited somewhat uniform immunolabeling density where they were immunopositive, Kv1.1 channel showed great variability in terms of immunolabeling density (Mayadali et al., 2021). We carefully interpreted this as possibly having varied protein expression to provide a range of excitability characteristics. However, in this study, we found also Kv3.1, HCN1 as well as Kv1.1 having not only inconsistent differences in MIF and SIF motoneuron immunolabeling, but also variability within the entirety of the trochlear nucleus (as opposed to only within SIF motoneuron population in monkey) (Figures 5, 6). For instance, as observed with Kv3.1 immunolabeling, MIF motoneurons expressed weaker immunoreactivity consistently (compared to SIF motoneurons) in some sections (Figures 5e, f), whereas in others this qualitative difference was not observed consistently, even seen for many SIF motoneurons (Figures 5e, f). Unfortunately, it was not within the scope of this study to rule out the possibility that these channels can be variably expressed in all motoneurons and are not differentially expressed in MIF vs. SIF motoneurons.

The variable immunolabeling of ion channel proteins, as well as PNs, therefore might coincide with previous observations indicating continuum in motor neuron types in terms of biophysiological properties (Davis-López de Carrizosa et al., 2011). Combination of this variety in motoneuron types with continuum in muscle fiber types and mitochondrial content could be a plausible explanation of achieving the eye movements with different sensitivities (Mayr, 1971; Kjellgren et al., 2003; Asmussen et al., 2008; McLoon et al., 2011).

#### 5.4.3 Internuclear neurons of the human abducens nucleus lack HCN1, in contrast to monkey

One striking difference between monkey and human INTs was found in HCN1 immunolabeling. In monkey abducens nucleus, INTs were found strongly immunolabeled for HCN1 similar to SIF motoneurons, whereas MIF motoneurons lacked HCN1 labeling (Mayadali et al., 2022). In human abducens nucleus, however, while SIF motoneurons mostly expressed strong HCN1 immunolabeling, INTs exhibited no immunoreactivity (Figures 6e, f). This was particularly interesting, as INTs are required to convey the horizontal eye movement signal to the contralateral oculomotor nucleus to generate synergistic activation of medial rectus motoneurons for conjugate horizontal eye movements. With small differences, INTs fire similarly to SIF motoneurons with burst-tonic pattern (Delgado-Garcia et al., 1977; Fuchs et al., 1988). Therefore, in the investigation of monkey INTs and SIF motoneurons, to have very similar immunolabeling of proteins related to intrinsic firing properties was congruent to their function (Mayadali et al., 2021). Indeed, monkey INTs showed very similar labeling with SIF motoneurons for ion channel and transmitter related proteins (Mayadali et al., 2021). Only qualitative differences in protein expression for monkey abducens INTs were slightly stronger Kv3.1 expression accompanied by slightly stronger PN ensheathment, as well as stronger NMDAR1 expression, which could be interpreted as compensatory variations, allowing INTs to fire slightly earlier and at a higher rate (Delgado-Garcia et al., 1977) to help synchronize SIF motoneurons of medial rectus and lateral rectus muscles. However, steep differences in HCN1 immunoreactivity for INTs in both trochlear and abducens nuclei was unexpected. Moreover, SIF motoneurons in human abducens also showed some variety in the immunolabeling intensity (Figure 6f), which was not observed in monkey abducens nucleus (Mayadali et al., 2022). This might indicate that SIF motoneurons could be regulating a range of post-inhibitory readiness for action potential, and resting membrane potential through HCN1 expression (Kase and Imoto, 2012).

HCN1 labeling was comparable in SIF and MIF motoneurons in trochlear nucleus, however, it was stronger in SIF motoneurons in the abducens nucleus. HCN2 labeling was comparable in SIF and MIF, and it was only punctate. There was putatively robust expression of HCN4 as evidenced by strong antibody binding in abducens and trochlear nucleus. Strong expression of fast acting HCN channel expression—such as HCN1 is suggestive of need for rapid kinematics, a fundamental requirement to generate post-inhibitory rebound. However, post-inhibitory rebound is generated in the burst neurons, which then project to the motoneurons (Enderle and Engelken, 1995; Shaikh et al., 2017; Mayadali et al., 2022). Hence, the latter provide readiness to keep high firing rate, but do not generate a burst. This requirement for bursting is served by higher expression of HCN1 in SIF motoneurons, along with robust expression of HCN4 along with other fast-acting ion channels. The rapid kinematics needed for saccades is less utilized in torsional system, hence trochlear nucleus lacks strong HCN1, compared to abducens nucleus. While vertical system is also served by the trochlear nucleus, latter is not the exclusive mode of driving vertical eye movements. In other words, readiness for rapid firing rate serving rapid kinematics is justified more so in the horizontal system (such as abducens) compared to trochlear nucleus. The study further supports the fact that SIF motoneurons are more likely to support rapid kinematics, such as saccades, in comparison to MIF motoneurons.

Furthermore, the HCN1 immunolabeling of motoneuronal somata and cell membranes in human compared to a strong cell membrane labeling in the monkey abducens and trochlear nuclei (Figure 6; Mayadali et al., 2022) could be due to differences in tissue fixation, since transcardial perfusion of monkey tissue allows for better preservation of protein localization.

Stronger HCN4 immunolabeling in putative INTs of trochlear nucleus, together with the decreased HCN1 isoform, suggested a shift in HCN channel subunit expression toward the subunit with the slowest kinetics (Kase and Imoto, 2012; Benarroch, 2013). As this was not clearly observed in the INTs of the abducens nucleus, biophysiological differences, as implicated in our present results and our study of monkey motor nuclei, should be further investigated (Mayadali et al., 2021).

In the human tissue, post-mortem delay, fixation protocol and paraffin embedding are known factors that degrade the quality of histochemical methodology. The immunoreactivity of antibodies that function well with mouse or monkey tissue specimen can often yield very low contrast stainings. And in the case of ion channel proteins, and qualitative comparison of protein expression levels, these detrimental effects disable an accurate reading of protein localization within neurons, or immunolabeling intensity (Gonzalez-Riano et al., 2017).

## 6 Conclusion

Histochemical investigation of neurons in human trochlear and abducens nuclei revealed three main conclusions: (i) there exists a motoneuron population that differ from previously characterized MIF and SIF motoneurons in terms of their perineuronal net expression. These motoneurons express weak PNs and lie between MIF and SIF motoneurons in terms of their diameters. (ii) Immunolabeling patterns of ion channel proteins observed in human trochlear and abducens nuclei revealed significant differences to the observations on these nuclei of rhesus monkey. Unlike MIF and SIF motoneurons of monkey trochlear and abducens nuclei, there are often no clear differences in immunolabeling of Kv1.1 and Kv3.1 channels, or HCN channels. Moreover, varied immunoreactivity was observed with multiple ion channel proteins. (iii) Internuclear neurons (INTs) of abducens (and trochlear) nucleus deviated from SIF motoneurons, especially with HCN1 channel immunolabeling, where SIF motoneurons were mostly strongly labeled and INTs were not. Such differences and prominence of HCN1 in SIF of abducens nucleus suggest readiness for high velocity horizontal saccades. The immunohistochemical examination of human tissue in the current study thus represents a major step in characterizing morphological and biochemical features of motoneurons that allow for the wide range of eye movements. It further allows definition of pharmacological properties, which in turn could provide a basis for any corresponding changes in human eye movement pathologies.

## Data availability statement

The raw data supporting the conclusions of this article will be made available by the authors, without undue reservation.

## Ethics statement

The studies involving humans were approved by the Ethics Committees of the Klinikum der Ludwig-Maximilians Universität. The studies were conducted in accordance with the local legislation and institutional requirements. The human samples used in this study were acquired from two brains donated to the Institute of Anatomy of the Ludwig-Maximilians Universität and one brain provided by the Institute of Legal Medicine of the Ludwig-Maximilians Universität. Written informed consent for participation was not required from the participants or the participants’ legal guardians/next of kin in accordance with the national legislation and institutional requirements.

## Author contributions

ÜM: Conceptualization, Data curation, Formal analysis, Investigation, Methodology, Validation, Writing – original draft, Writing – review & editing. CC: Data curation, Formal analysis, Investigation, Methodology, Validation, Visualization, Writing – review & editing. IS: Methodology, Resources, Writing – review & editing. AS: Conceptualization, Funding acquisition, Investigation, Writing – review & editing. AH: Conceptualization, Data curation, Formal analysis, Funding acquisition, Investigation, Project administration, Supervision, Validation, Writing – review & editing.
